# The Impact of Chloride and Sulphate Aggressiveness on the Microstructure and Phase Composition of Fly Ash-Slag Mortar

**DOI:** 10.3390/ma14164430

**Published:** 2021-08-07

**Authors:** Paweł Falaciński, Agnieszka Machowska, Łukasz Szarek

**Affiliations:** Faculty of Building Services, Hydro and Environmental Engineering, Warsaw University of Technology, Nowowiejska St. 20, 00-653 Warsaw, Poland; pawel.falacinski@pw.edu.pl (P.F.); lukasz.szarek@pw.edu.pl (Ł.S.)

**Keywords:** slag-fly ash binder, CFBC fly ash, circular economy, chloride aggressiveness, sulphate aggressiveness

## Abstract

The article discusses the results of examining the impact of aggressive solutions on specimens of mortars with a slag-ash binder. Bar specimens were exposed to unidirectional diffusion of sodium chloride and sodium sulphate for 90 days. Next, the specimens were subjected to flexural and compressive strength tests, ion content tests, XRD phase composition tests, and microstructural SEM-EDS tests. The test results indicated that aggressive solution action resulted in decreased flexural strength, however, it did not impact the compressive strength of mortars. A minor impact of chloride ions on the pH of the pore liquid was recorded, while the tests did not show any influence of sulphate ions. Furthermore, aggressive ion concentration decreased in deeper specimen slices. Specimen phase composition testing after chloride ion action indicated the presence of a small amount of Friedel’s salt, while regular sodium chloride crystals were identified in the microscopic image. The performance properties of mortars exposed to the action of aggressive solutions were maintained.

## 1. Introduction

One of the key issues in EU countries are the millions of tons of coal combustion by-products (CPBs) generated each year [1] and millions more accumulated in landfills. Utilizing CPBs in line with the ideas of Circular (zero-waste) Economy (CE), in economy branches such as civil engineering, road construction, agriculture, or production of plastics is not always possible due to the particular properties of such waste. According to current data [1], only 25% of the CPBs are re-used, satisfying CE principles. Ashes from coal combustion in fluidized-bed boilers are particularly difficult in terms of reusing. A different crystallographic structure increased (relative to conventional ash) content of calcium compounds and unburned coal components, as well as high water demand, significantly restrict the application-related potential of this waste.

Composites made with the addition of combustion by-products should be characterized by sufficiently high durability, apart from their mechanical properties. The presence of sulphates in the external environment, e.g., groundwater, is particularly dangerous in terms of material durability. Indeed, the sulphate corrosion process in concrete is relatively well studied [2,3]. The resistance of concrete to aggressive waters with sulphates may be achieved by using hydraulic and pozzolanic additives [4,5]. The impact of pozzolanic additives involves decreasing the Ca(OH)_2_ value and increasing the share of the CSH phase in the paste, which significantly lowers the content of large capillary pores (mesopores) [6].

Chloride corrosion is equally dangerous to construction composites. Corrosion tests of cement composites in high-concentration chloride solutions showed that the corrosion process started with shrinkage, which results from the thickening of the C-S-H gel due to osmotic pressure. This leads to the formation of microcracks that are pathways for the rapid diffusion of chlorides inside the material structure [7]. Chloride ions in a cured cement mortar are found in two phases, namely, expansive alkaline calcium chloride (Ca(OH)_2_·CaCl_2_·H_2_O) and Friedel’s salt (CaO·Al_2_O_3_·CaCl_2_·10H_2_O). The chloride expansion process results from increased solid phase volume during the formation of Ca(OH)_2_·CaCl_2_·H_2_O. In many cases of structural chloride corrosion, the presence of an alkaline calcium chloride was identified in the mortar-aggregate contact zone [8]. With regard to reinforced concrete structures, the basic outcome of chloride aggression is, however, reinforcing steel corrosion. Corrosion is initiated under a specific chloride ion concentration, and the total chloride content does not determine the progress of steel corrosion in concrete. This is because only free chloride ions react chemically, leading to steel corrosion. The free chloride content in non-carbonated concrete amounts from 5% to 23% of their total content [9] and depends on, e.g., cement type, used additives, etc. Chloride corrosion is supported by sulphate aggression, leading to Friedel’s salt decomposition and chloride ion release. The decrease in pH, associated with the carbonation of cured concrete, also accelerates the chloride corrosion process.

This article attempts to evaluate the impact of liquids containing chloride and sulphate ions on the phase composition and microstructure of mortars with a clinker-free fly ash-slag binder.

## 2. Materials and Methods

### 2.1. CFBC Fly Ash

Fluidized bed brown coal combustion fly ash is generated in circulating fluidized bed boilers, where the combustion process takes place at atmospheric pressure (CFBC—Circulating Fluidized Bed Combustion). It is composed of grains with developed specific surfaces, which leads to the high-water demand for this type of ash. The ash is characterized by pozzolanic and/or hydraulic properties. It is mainly used to fill post-mining headings, in road construction, for soil stabilization and reclamation and deacidification or neutralization of acidic industrial wastewater (due to the high pH resulting from increased calcium compound content). Fluidized-bed fly ash is not approved for use in cement or concrete production; it can only be applied as a secondary mineral additive for concrete, in an amount below 5%.

The fluidized-bed brown coal combustion fly ash (from “Turów” Power Plant, Bogatynia, Poland) used in the experiment to make up a test mortar was investigated with the laser diffraction method to determine the particle size distribution. The results were presented in [10]. The CFBC fly ash consisted of grains of diameter below 85 µm in 90% of the total specimen mass. Moreover, up to 10% of the total mass included grains of diameter up to 3.73 µm, and up to 50% consisted of grains of diameter up to 24.37 µm [10]. The fineness of the fly ash grains tested using the wet sieving method (also noted by [11]) was equal to 28.2% of the total mass. The Blaine’s specific surface tested with the air permeability method along with [12] was equal to 4690 cm^2^/g. Water demand investigated with flow table method along with [13] equalled 125.4%.

The CFBC fly ash chemical composition was presented in [14]. The fly ash primarily contained calcium oxide at 21.10%, silicon oxide at 34.87%, and aluminium oxide at 23.09%. The content of free calcium oxides was 7.0%, and 13.9% for reactive calcium oxide. Reactive silica content was equal to 19.5%. Sulphur oxide content was 5.66% and 0.04% for chlorides [14]. The high content of free calcium oxide in fluidized-bed fly ash was not a threat to the constancy of the volume of composites it made up, since this ingredient is very active and undergoes a rapid chemical reaction in contact with water.

The CFBC fly ash examined with the X-ray diffraction method was found to also contain anhydrite, quartz, calcite, and hematite, and it held its composition in a disordered structure in the semi-amorphous phase, due to dehydration and dehydroxylation of the gangue (i.e., metakaolinite), raising the background for the diffraction intensity for the range of ~25–40° (2θCu_Kα_) [14,15,16]. The recorded minor amount of calcium hydroxide indicated slight hydration of free calcium oxide [14]. The free CaO and anhydrite content activated the hydration of composites with blast furnace slag. Fluidized-bed brown coal ashes carry silicate and calcium ions that participate in the formation of such phases as ettringite and hydrated calcium silicates.

### 2.2. Ground Granulated Blast Furnace Slag

Blast furnace slag is a material with latent hydraulic properties that does not bind with water or binds very slowly. The slag hydration process depends on the glassy phase, an appropriate degree of grain grinding, and their activation. The necessity to apply blast furnace slag binding reaction activators results from the fact that a poorly permeable layer of aluminium silicates is formed on the surfaces of grains in contact with water, which limits water penetration inside the grains, hence stopping the hydration process [17].

Composites made of ground granulated blast furnace slag demonstrate high resistance to chloride and sulphate ions aggressiveness due to the slag’s properties. The products of slag hydration fill up the microstructure of composites, thus increasing its tightness. The C-S-H phase develops in pores, while a lower amount of Ca(OH)_2_ forms hydrated composites. This outcome results in higher resistance to corrosive ions diffusion when compared with Portland cement composites [18].

The slag used in the experiment was investigated with the laser diffraction method to determine the particle size distribution. The results were presented in [10]. The slag was found to contain grains in the range 0.34–89 µm. Grains below 35.7 µm made up 90% of the total investigated specimen mass, 50% of grains were found to have a diameter below 14.93 µm, and up to 10% were grains of diameter below 2.63 µm [10]. The fineness of the slag’s grains, tested with the wet sieving method along with [11], equalled 2.3% of the investigated specimen mass, and Blaine’s specific surface tested with the air permeability method along with [12] was 3150 cm^2^/g.

The chemical composition is significant in terms of slag activity. It was presented in [14]. The primary ingredients of slag used for the tests consisted of CaO (total: 45.15%), SiO_2_ (36.27%), Al_2_O_3_ (9.15%) and MgO (5.86%), while its secondary ingredients were Fe_2_O_3_ (1.55%), Na_2_O (0.01%), K_2_O (0.05%), P_2_O_5_ (0.69%). The slag activity coefficient, determined in accordance with PN-EN 197-1 [19], was 1.41, and the content of basic oxides was 90%. This means that the studied slag is highly basic, which ensures an appropriate level of its activity. The high glassy phase content in the slag was also confirmed by IR tests [14].

As an activator for the hydration of ground blast furnace slag, the authors used CFBC fly ash—a by-product of combustion with a high alkaline reaction (solution pH: 12.7). No chemical activators were added. The tests on pastes with a fly ash-slag binder in the early and later hydration stages confirm the potential of a clinker-free binder [14,20].

### 2.3. Specimen Preparation

The mortars for studying the chloride and sulphate aggression were prepared in the form of 40 × 40 × 160 mm bars, using the same methodology as for standard mortar, in accordance with the provision of the procedure in [21]. The mortar composition is given in Table 1.

Until demoulding, the specimen was cured for 48 h in a steel mould and under a foil cover, in a room with an ambient temperature of 20 ± 2 °C. Next, the bars were removed from the moulds and placed in a bath with water at a temperature of 20 ± 2 °C, where the specimens were cured until the scheduled test date.

**Figure 1:**
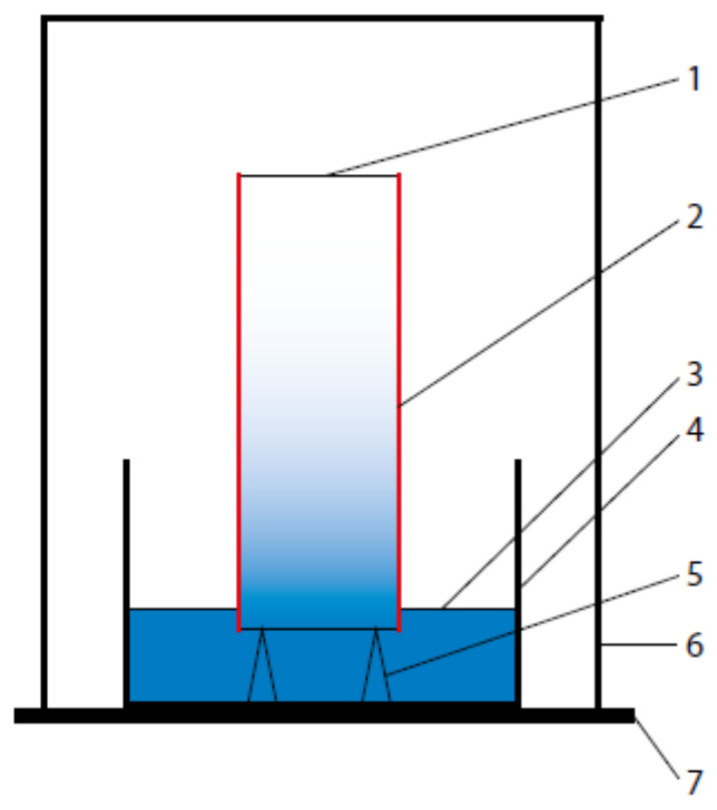
Transient state aggressive ion diffusion test diagram; 1—tested specimen; 2—protecting coating (epoxy resin); 3—penetrating solution; 4—vessel with penetrating solution; 5—specimen supports; 6—protecting cover; 7—base.

In order to prepare the specimens for exposure to the aggressive chemical action of aqueous solution (NaCl and Na_2_SO_4_), the authors followed the procedures set out in [22]. After 28 days of curing, the mortars were exposed to 90 days of aggressive solution action through the unidirectional diffusion method. For this purpose, the slag-fly ash mortar bars were removed from the water, and then, in order to enable one-dimensional chloride/sulphate diffusion, their surfaces were coated with two layers of epoxy resin (through painting). After the resin had dried, an approx. 10 mm thick layer was cut from one side and disposed of. The remaining specimen was placed in a 16.5% NaCl aqueous solution and in a 1.0% Na_2_SO_4_ aqueous solution, so that the exposed specimen surface was immersed in the solution, at a depth of 10 mm. The aggressive ion diffusion test diagram is shown in Figure 1. After 90 days of exposure to the corrosive action of aggressive sulphate and chloride ions, the specimens were removed from containers and cut (dry) into approx. 1.0 cm thick slices.

### 2.4. Test Methods

The following laboratory property tests were conducted:

The flexural and compressive strength of the specimens was tested as per standard [21]; the specimens were stored in tap water, at a temperature of 20 ± 2 °C, in a solution of chloride and sulphate ions of the same temperature. After the curing time had elapsed, the specimens were taken out of the water/solution, dried with a paper towel, measured, weighed, and subjected to flexural and compressive strength testing.

- The chloride and sulphate ion contents were assessed as per standard [23]. The test procedure set out in the standard defines transient state diffusion, since the stream of diffusing aggressive ions changes over time. As the changes can be induced by the binding of ions in the material, fragments of previously cut mortar slices were ground separately in a porcelain mortar, and the wet chemical analysis was applied [23]. Then the content of chloride ions (for 6 slices) and sulphate ions (for 4 slices) for each of the mortar slices exposed to the action of NaCl and Na_2_SO_4_ was determined. It was noted that the analysis results were related to the binder content in the mortars, taking into account the natural moisture of the specimens.
- The sulphate content results were then converted into SO_4_^2−^ content. Furthermore, water extraction as per [24] was executed for each of the slices (6) of each specimen, after previously grinding and sieving the material through a 1.0 mm mesh-sized filter. Water extracts were used as a mortar pore liquid model, where the pH was determined.

The phase composition was studied by applying the XRD method on powdered specimens, by employing a Bruker D8 Advance device equipped with a position-sensitive LYNXEYE detector operating over Bragga-Brentano geometry, using CuKα (λ = 0.15418 nm) radiation with a nickel filter. The measurements were recorded over an angular range 2θ from 8° to 75°, with an increment of 0.03° and a recording time of 960 s/increment.

The microstructure was studied by means of a ZEISS LEO 1430 scanning electron microscope equipped with an Oxford ISIS 300 (Oxford Instruments) energy dispersion detector (EDS). The investigated small test coupons consisted of specimens from inside the bars that were dried at a temperature up to 40 °C, so that there were no chemical reactions that could lead to the reaction of some products found in the specimen’s microstructure. After drying, the specimens were placed on a stand and gold sputter coated. The observations were conducted under low vacuum conditions (6 × 10^−5^–7 × 10^−6^Torr), and a voltage of 20 kV (80 μA).

## 3. Results and Analysis

### 3.1. Flexural and Compressive Strength

**Figure 2:**
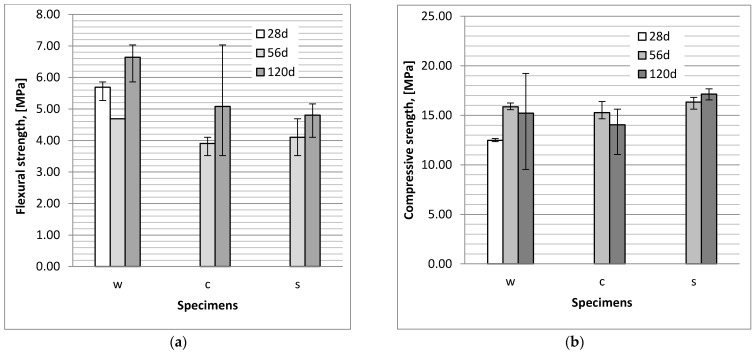
Flexural (**a**) and compressive (**b**) strength of slag-fly ash binder bars; w—specimens stored in tap water, c—specimens stored in a NaCl solution, s—specimens stored in a Na_2_SO_4_ solution.

On analysing the flexural (Figure 2a) and compressive (Figure 2b) strength test results for the bars stored in water and aggressive solutions, we observed that these parameters increase with curing time. Sulphate and chloride ions were also found to have a minor impact on the flexural strength of the studied specimens. Compared to the specimens stored in water, the average value of the strength of specimens exposed to chloride aggressiveness was lower by 16%, and in the case of specimens exposed to sulphate aggressiveness—by 13%. Beyond the aforementioned, the flexural strength of both specimens grew over time, although the growth is noted within error limits. On analysing the results of the compressive strength, we recognized a difference in the values in the case of 120 days old specimens (after 90 days of exposure)—this parameter was lower than the strength values for specimens cured in tap water. We also observed that in the case of specimens stored in chloride solution, the compressive strength was lower than that of the specimens stored in a sulphate solution. Comparing to the values of strength of specimens stored in tap water it can be noted that the sulphate solution seemed to have very low or even no impact on specimen compressive strength.

### 3.2. Chloride and Sulphate Ion Content

**Figure 3:**
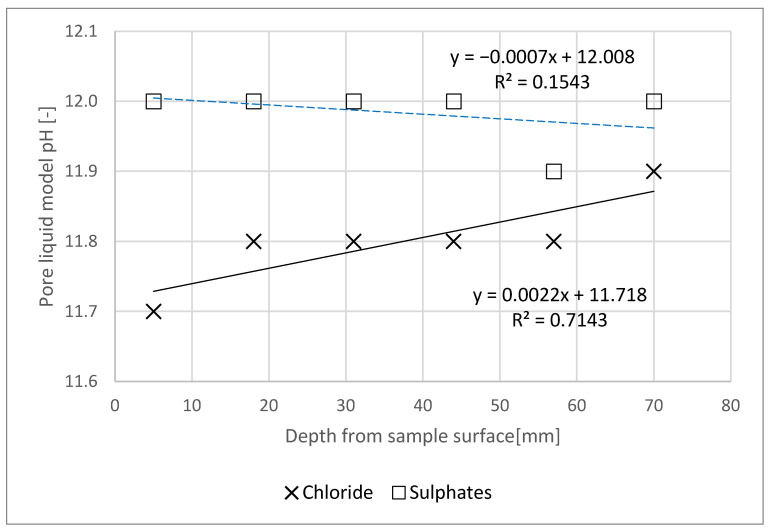
Pore liquid model reaction change profile relative to specimen depth.

**Figure 4:**
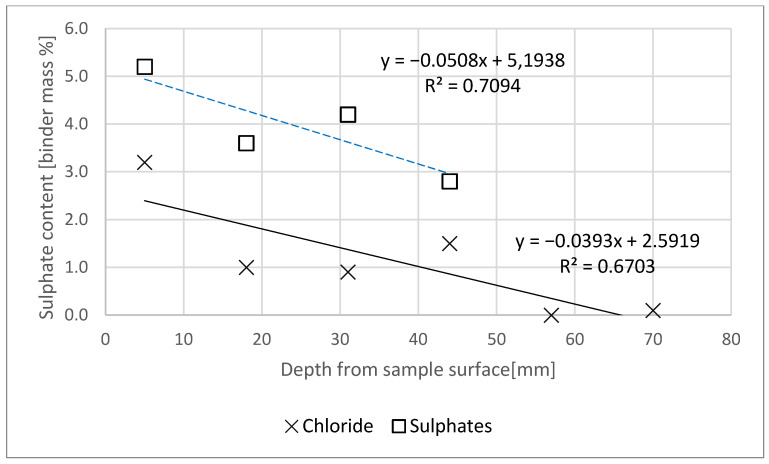
Chloride and sulphate ion content profile per specimen depth, relative to binder weight.

Figure 3 shows the change in the reaction of the pore liquid model in specimens slices relative to the depth from the surface of the specimen exposed to the action of a NaCl and Na_2_SO_4_ solution. Figure 4 reveals the chloride and sulphate ion content in slices relative to the binder content, depending on the depth from the surface of the specimen exposed to the action of a NaCl solution. The horizontal axis on both figures represents the depth stipulated as the distance of the thickness centre point in the mortar slice in question, relative to the specimen’s plane of contact with the solution, considering saw blade thickness.

The action of chlorides led to a minor pH decrease towards the plane of the specimen exposed to the solution (significant r-Pearson correlation = 0.971). This slight difference can be explained by the fact that chlorine diffusion as NaCl, can react to raise the pore liquid pH, since Cl^−^ is replaced with OH^−^, which results in the formation of NaOH, thus, the pH does not have to drop significantly due to diffusing reactions [25,26]. This may indicate that NaCl has a lower chemical aggressiveness relative to, e.g., NH_4_Cl [27]. Water extract pH at a depth of already approx. 20 mm was at the limit value of pH = 11.8—below a level in which the first symptoms of reinforcement steel passive layer decomposition appear [28].

**Figure 5:**
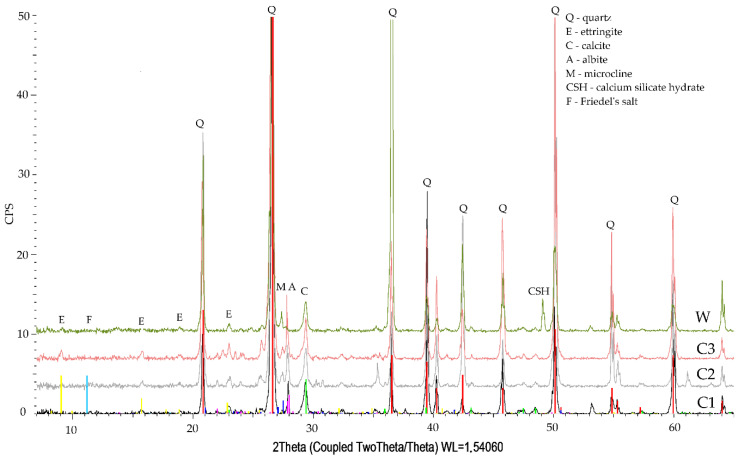
Diffraction pattern of specimen slices No. 1, 2, 3 (C1, C2, C3) after chloride ion diffusion, against the background of the specimen stored in tap water (W).

Chloride concentration in the specimen, relative to the binder mass decreased together with increasing specimen depth (Figure 4). The requirements set out in [29] apply only to chloride ion content in concrete, relative to cement content, which is determined by summing the chloride content in individual concrete ingredients (environmental impact is not taken into account). The maximum chloride content in concrete without reinforcement is 1.0% of the cement weight. After 90 days of one-dimensional NaCl diffusion, the percentage concentration of the ions in question was lower (Figure 4) than the limit value in a specimen at a depth below 60 mm, and even below 50 mm, as a prediction based on the trend line. XRD tests indicated the presence of Friedel’s salt for only one of the specimens, and probably only a small amount of it (Figure 5). This, we believe, resulted from the pore liquid model pH, since Friedel’s salt is decomposed at pH < 12 [30,31]. Furthermore, the source literature contains the information [25,32] that the formation of Friedel’s salt will be limited by the total available Al content in the binder, and not by the very content of AFm phases, since all aluminate hydrates will be transformed at a similar range of concentrations as Friedel’s salt.

As a result of increased sulphate ion concentration in cement matrices, the pH of the pore liquid decreases gradually [33]. In the case of the studied specimens, there was no observed pore liquid model reaction change due to the Na_2_SO_4_ action that might be caused by the immersion in a NaOH solution, as in the case of chlorides. The chemical reaction of the pore liquid model throughout the entire specimen depth (pH_min_ = 11.9) was more alkaline than pH = 11.5—the level below which C-S-H phase deliming increases very rapidly [34], and also above pH = 11.8, wherein first symptoms of reinforcement steel passive layer decomposition appear [28,35].

Sulphate concentration in the specimen, relative to the binder mass, decreased together with increasing specimen depth (Figure 4). A permissible sulphate content in concrete is 0.5% (relative to concrete weight) [36], which is a value at a level of approx. 2.25% relative to the binder weight, after conversion for the specimens in question. All 4 studied specimen slices were characterized by concentrations higher than the limit value (Figure 4), however, based on the obtained results, it can be predicted that the limit value would be probably exceeded at a depth of 58 mm.

**Figure 6:**
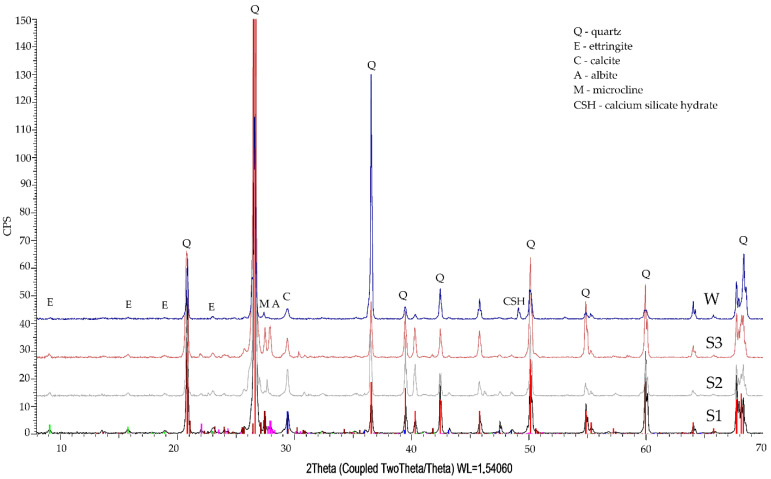
Diffraction pattern of specimen slices No. 1, 2, 3 (S1, S2, S3) after sulphate ion diffusion, against the background of the specimen stored in tap water (W).

The impact of Na_2_SO_4_ on the cement matrix primarily involved reacting with Ca(OH)_2_ and the formation of gypsum and sodium hydroxide [37,38,39,40,41], while specimen diffraction pattern graphs (Figure 6) did not indicate the presence of portlandite or gypsum. Gypsum could react with calcium aluminate hydrate [42] to form ettringite—which the reflections may, however, indicate. There are also reflections indicating minerals similar to albite and also the C-S-H phase.

Despite the average corrosive activity of sodium cations [37], they are considered particularly dangerous to concrete with reactive aggregate [43].

### 3.3. Phase Composition Qualitative Tests

The phase composition XRD tests of the specimens stored in tap water and exposed to the action of aggressive solutions indicated the presence of quartz, calcite, calcium aluminosilicates, and ettringite in all studied specimens (Figure 5 and Figure 6).

Specimens after exposure to chloride aggression (Figure 5) revealed the presence of a slight amount of Friedel’s salt (reflections at approx. 11°) in the first slice that was in direct contact with the aggressive solution. The reflections of this phase disappear in subsequent slices. Visible reflections at approx. 29° and 49° confirm the presence of hydrated calcium silicate phases in the specimens, and reflections at 9°, 16°, 19°, and 23° are responsible for the presence of ettringite in the specimens and are more intensive in internal slices of the specimens (No. 2 and 3) relative to the slice in direct contact with the aggressive solution.

Specimens exposed to the action of sulphate aggressiveness exhibit intensive reflections originating from quartz, ettringite, and calcite, as well as hydrate aluminosilicates (Figure 6). The reflections from quartz are less intense than the reflections in specimens exposed to chloride ions aggressiveness. 

### 3.4. Microstructure

**Figure 7:**
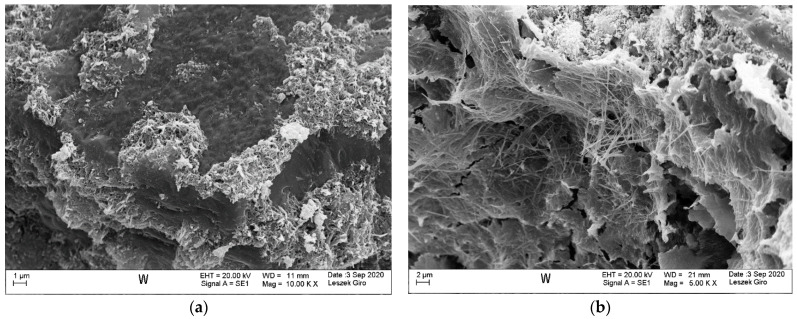
Microstructure of the specimen stored in tap water; visible compact C-S-H phase (**a**) and fibrous C-A-S-H phase formations (**b**).

Microstructure tests were conducted on slices cut from specimens stored in tap water and exposed to the action of a NaCl and a Na_2_SO_4_ solution (1 cm thick slice in direct contact with the solution), in order to identify potential products formed after the solutions diffusing inside the specimens. Mortar specimens cured in tap water were characterized by a compact, tight structure, without visible microcracks within the analysed area. The hydrated calcium silicate phase appears in the form of a poorly crystallized compact structure and radially emerging needles. We also saw locally observable coalescing fibres forming a “honeycomb” C-S-H phase. In addition, we noted that the hydrated aluminosilicate phase occurs in the form of long fibres growing out in voids, bundling together to form a compact structure. The C-S-H phase contained individual ettringite crystals in the form of hexagonal columns. The microstructure also exhibited clusters of massive CaCO_3_ crystals and clayey forms—in the guise of thin-layered plates (Figure 7).

**Figure 8:**
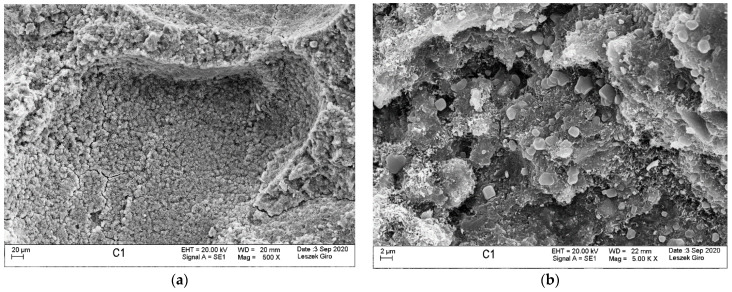

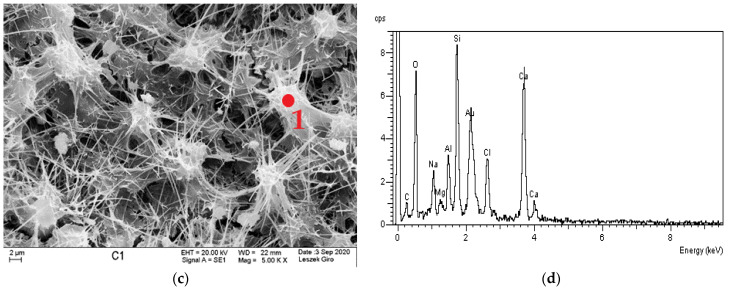
Microstructure of the specimen exposed to chloride ions; visible cracks in the C-S-H phase (**a**); irregular NaCl crystals in the C-S-H phase (**b**); hydrated aluminosilicates (**c**) and analysis in micro-area point 1 (**d**).

The specimens exposed to NaCl are more porous and are characterized by a higher number of microcracks relative to the specimens exposed to the action of Na_2_SO_4_ or cured in water. Bundles of thin and flat fibres growing into voids within the structure of the specimen can be noticed, as well as individual sodium chloride crystals dispersed throughout the structure, which have morphologically taken a regular form (Figure 8b). The EDS analysis of the studied specimen indicates that chloride ions had been incorporated into the mortar microstructure (Figure 8c,d). This phenomenon can have a negative impact on mortar properties, since the stresses generated during sodium chloride crystallization exceed the concrete tensile strength, which might lead to the formation of microcracks in its structure [44,45]. It should be remembered, however, that the composition of the binder used to produce the composite has the greatest impact on chloride ions binding within the matrix. The studies by [46] indicate that binders with a large slag content (30–60%) are least susceptible.

**Figure 9:**
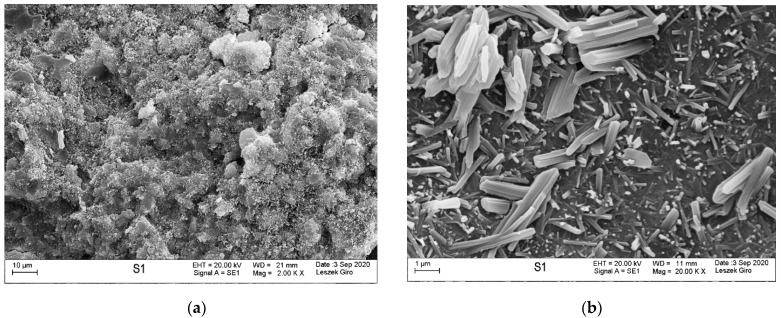

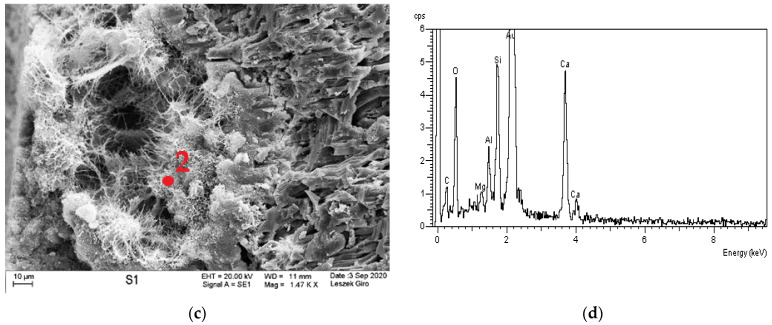
Microstructure of specimens exposed to the action of sulphate ions; C-S-H phase in compact and acicular form (**a**); ettringite columns of pseudo-hexagonal habit (**b**); fibrous forms of hydrated calcium aluminosilicates and irregular CaCO_3_ crystals (**c**) and analysis of fibrous forms in micro-area point 2 (**d**).

A slice of the specimen exposed to the action of Na_2_SO_4_ reveals that it is characterized by a compact structure, with individual cracks within the observed area. Moreover, the C-S-H phase had taken a compact form and the form of radially arranged and clustered needles (Figure 9a). Also, locally present radially arranged ettringite columns of pseudo-hexagonal habit and capillary fibres filling the voids, while bundling was observed (Figure 9b). Furthermore, CaCO_3_ in the form of irregular sheared crystals with sharp tips was noted in the specimen (Figure 9c,d). In addition, hydrated calcium aluminates in the form of hexagonal plates were locally present. This came about due to the CFBC fly ash used to make the binder.

## 4. Conclusions

When analysing the results of conducted tests involving mortars with a slag-fly ash binder that were subjected to unidirectional diffusion of NaCl and Na_2_SO_4_ solutions, the following conclusions can be drawn:

1. The action of a NaCl and Na_2_SO_4_ solution caused a reduction of flexural strength of bars in comparison to the strength of specimens stored in tap water, probably due to the microcracks which were observed in the specimens.
2. The impact of chloride and sulphate ion diffusion on the compressive strength of mortars is minor and within measurement uncertainty limits. The compressive strength of the specimens exposed to chloride aggressiveness is lower than the strength of specimens exposed to sulphate aggressiveness.
3. Chloride action had a slight (adverse) influence on the pH of the pore liquid. However, no impact of sulphate action of the pore liquid pH was noted—this outcome may be due to its formation in a NaOH solution.
4. Chloride and sulphate concentration in the specimen decreased with increasing specimen depth.
5. Based on the tests, it can be concluded that the content of chlorides and sulphates in the specimen was lower than the limit concentration at a distance of less than 60 mm from the specimen’s plane of contact with the aggressive medium.
6. The qualitative tests of phase composition using the XRD method revealed minor amounts of Friedel’s salt in the specimen exposed to the action of chloride ions.
7. The diffraction patterns for all studied specimens exhibit the clear presence of quartz, calcite, calcium aluminosilicates, and ettringite. The presence of a small amount of Friedel’s salt was recorded in specimens subjected to diffusion with chloride ions.
8. SEM-EDS microstructure tests indicated that there were more microcracks on the surface of specimens subjected to ion diffusion than the surface of other specimens. Moreover, sodium chloride in the form of regular crystals was evident. In addition, chlorine ions were incorporated into the hydrated calcium aluminosilicates.
9. The local presence of ettringite crystals in the form of pseudo-hexagonal columns were identified in the microstructure of specimens exposed to sulphate ion diffusion.

The specimens of fly ash-slag mortars exhibit tight microstructure because of the main component—blast furnace slag. The products of hydration fill in the free spaces and pores thus tightening the structure and limiting the aggressive ions diffusion in the specimen.

## Figures and Tables

**Table 1:** Mortar composition.

No.	Ingredient	Ingredient Content [g]
1	Water	225
2	GGBFS	315
3	CFBC fly ash	135
4	Standard sand	1350
5	w/b ratio	0.5

## Data Availability

The data presented in this study are available on request from the corresponding author.
